# Long-Term Survival and Causes of Death in Patients below the Age of 60 with Traumatic Spinal Cord Injury in Germany

**DOI:** 10.3390/jcm11010026

**Published:** 2021-12-22

**Authors:** Roland Thietje, Birgitt Kowald, Ralf Böthig, Arndt P. Schulz, Markus Northmann, Yannick Rau, Sven Hirschfeld

**Affiliations:** 1 Center for Spinal Injuries, BG Klinikum Hamburg, 21033 Hamburg, Germany; b.kowald@bgk-hamburg.de (B.K.); r.boethig@bgk-hamburg.de (R.B.); schulz@biomechatronics.de (A.P.S.); m.northmann@bgk-hamburg.de (M.N.); s.hirschfeld@bgk-hamburg.de (S.H.); 2 Department of Biomechanics and orthopedic Research, University Lübeck, 23538 Luebeck, Germany; 3 Medical Faculty, University of Lübeck, 23538 Lübeck, Germany; yannick.rau@student.uni-luebeck.de

**Keywords:** spinal cord, spinal cord injuries, mortality, life expectancy

## Abstract

To study the mortality, cause and risk indicators of death in German patients with traumatic spinal cord injury, patients with traumatic spinal cord injury admitted to Berufsgenossenschaftliches Trauma Hospital Hamburg between 1 January 1997 and 31 December 2018, aged between 16 and 60 with a minimal survival of one year after injury, were included. Further criteria were the absence of life-limiting comorbidities at the time of injury. 223 deceased patients with traumatic spinal cord injury were identified, investigated on and partly compared to the surviving subjects. We aimed to discover specific complications that were related to Spinal Cord Injury and responsible for a possibly limited life expectancy. Data collection was performed during in- and outpatient treatment. A statistical analysis was performed to compare groups. The post-injury life expectancy was 25.0 years with a significant correlation regarding the level of lesion and severity of injury. The leading causes of death were cardiovascular diseases and pneumonia. Bladder cancer was the most common fatal malignant tumor. The life expectancy of patients suffering from traumatic spinal cord injury is limited. The longer a patient survives after injury and the lower the level of lesion, the more likely an age-related cause of death becomes. Bladder cancer is significantly more frequent when compared to the overall distribution of tumor diseases in Germany.

## 1. Introduction

The prevalence rate of traumatic spinal cord injury (SCI) in Germany is about 500 per million inhabitants, and the incidence rate amounts to 13 per million [1]. The optimization of rescue services and intensive care in combination with improvements in surgery and rehabilitation has contributed to an increased expectation of life after SCI [2]. Despite these improvements, tetraplegic patients still have a limited life expectancy compared to the normal population [3,4]. As an explanation for this fact, lung diseases, cardiovascular diseases and acute pneumonia have been described as the leading causes in the past [5]. Suicide has been described as a major factor, but in most studies, although it was elevated in comparison to the general population, it was not a significant cause of death [6,7,8,9,10,11].

In studies of our institution, the development of bladder cancer in SCI patients was clearly elevated in comparison to the general population. Specific reasons for this have yet to be determined, as indwelling catheters may be waived as a possible reason and other explanations are part of current investigations [3,12,13].

Although there is some recent retrospective data available from different national-based research projects, these are mainly retrospective descriptions, none of them covering the situation in Germany [8,14,15,16]. We therefore conducted this registry-based study to explore life expectancy and mortality causes in a large cohort, covering the situation in the north of Germany. We expect to discover a limited life expectancy in relation to the location and severity of SCI and we evaluate specific complications related to SCI that are responsible for this.

## 2. Materials and Methods

Since 1 January 1997, every new case of SCI (*n* = 7029) and every re-admission (*n* = 15,435) of an SCI patient was included in our SCI-Unit registry (Filemaker^®^, Claris International Inc., Cupertino, CA, USA). The first recorded injury occurred in 1951. Documentation consists in the collection of general epidemiological data, functional data (e.g., American Spinal Injury Association (ASIA) impairment scale), additional findings regarding SCI-related problems as well as SCI-independent diseases of importance [17,18].

From this registry, all cases of first presenting traumatic SCI between 1 January 1997 and 31 December 2018 over the age of 16 with a survival of more than one year after injury were identified before applying exclusion criterias (*n* = 2435).

**Figure 1:**
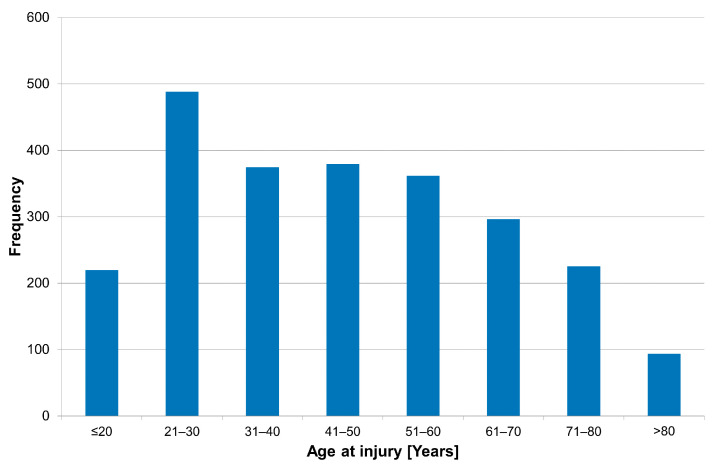
Age distribution of patients admitted with a new traumatic SCI (*n* = 2435).

The age distribution showed a large cohort in the group of 16 to 30 year old patients with declining numbers toward a higher age (see Figure 1).

**Figure 2:**
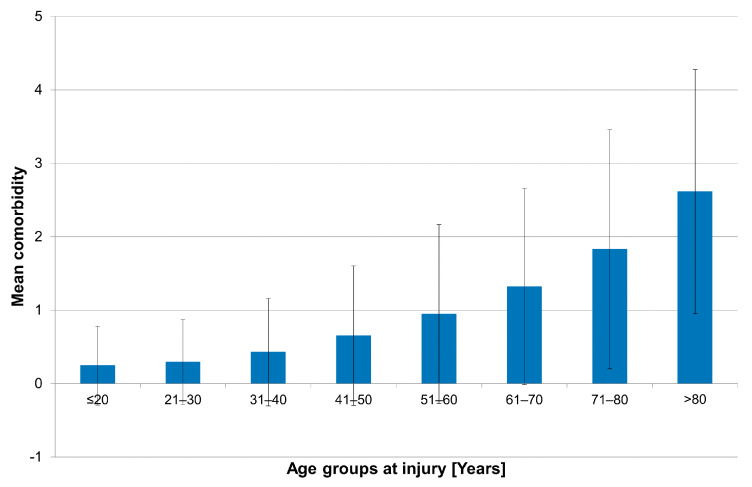
Bar graphs counting the number of comorbidities according to age groups at the time of injury.

A total of 539 cases of death occurred until 31 December 2018. In this cohort, we observed that unrelated comorbidities at the time of injury increased markedly after the age of 60 (see Figure 2). We therefore decided to cap this study, only including patients up to the age of 60 years at the time of injury. We attempted to reduce the impact of comorbidities that were already in existence at the time of injury.

**Figure 3:**
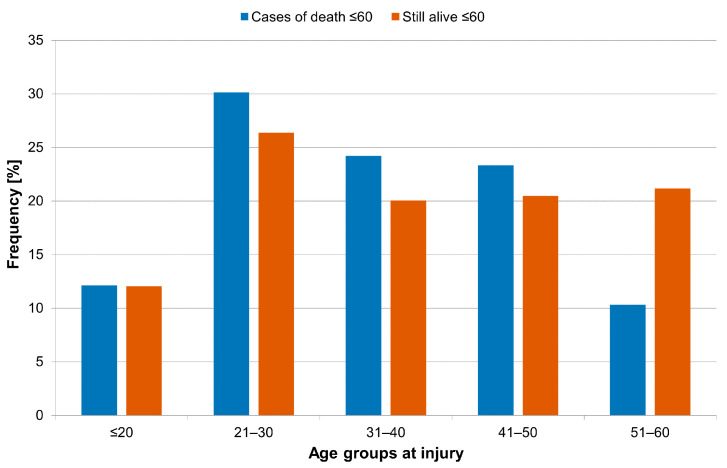
Age distribution of the study cohort (cases of death ≤ 60) of patients that died during follow-up compared to patients that were still alive (still alive ≤ 60).

The study population therefore only included patients who at the time of injury were 60 years and younger, survived for a minimum of one year after injury and did not reveal any life-limiting comorbidity at the time of injury. The pre-selection was established in order to prevent a distortion of the evaluation. 223 mortality cases meeting the above-mentioned criteria were detected. The groups of patients that were either deceased or still alive are depicted in Figure 3.

For the statistical analysis, the software SAS 9.2 (SAS Institute Inc., Cary, NC, USA) was used, which allows the management and statistical analysis of data.

Descriptive statistics such as the frequency scales, the averaging calculation, the determination of the minimum and maximum, and the standard deviation were used. Applying Shapiro–Wilks testing, normal distribution values were confirmed. To compare outcomes between two independent groups that were not normally distributed, the Wilcoxon Rank Sum Test was used. To test more than two groups in the presence of non-normally distributed data, the Kruskal–Wallis test was performed.

The level of significance for all statistical testing for this study was determined as *p* < 0.05. A chi-square test was conducted to show the dependence between qualitative characteristics.

All graphs in this paper were produced using SAS 9.2 software (SAS Institute Inc., Cary, NC, USA). All other graphics were drawn using Microsoft^®^ Excel (Microsoft, Redmond, DC, USA).

## 3. Results

### 3.1. Age at Injury and Etiology of Traumatic SCI

The study population included 223 patients. The gender distribution was 91.5% (*n* = 204) male, and 8.5% of the patients were female (*n* = 19). SCI occurred at an average age of 34.3 years. A comparison with the surviving population (*n* = 1598) showed an underrepresentation of female patients in the mortality group (8.5 vs. 19.3%, *p* < 0.01, Chi-square test).

No significant differences between men (34.2 years) and women (34.8 years) were observed regarding the mean age at injury.

Table 1 shows the degree of injury as defined by the ASIA Impairment Scale Type in both groups.

Table 2 shows the overall distribution of causes of TSCI in the study group. The most common cause was falls (52.9%), followed by transport accidents (35.0%), accidents caused by sports and leisure (7.3%), swimming accidents (4.2%)—most notably, diving into shallow water made up more than 50% of the sports and leisure causes. Suicide attempts made up 3 of 4 “other causes” of TSCI.

### 3.2. Life Expectancy

As seen in Table 3, life Expectancy significantly differs between levels of lesion (Kruskal–Wallis Test, *p* < 0.001). Assessing the neurological level of lesion, patients with a high level of lesion (C1–C4) Type A–C survived an average of 17.3 years (range 1.1–43.4), whereas tetraplegics with lower-level lesions (C5–C8) Type A–C survived for an average of 22.3 years (range 4.0–51.9). The Wilcoxon rank sum test was statistically significant (*p* = 0.0320). In paraplegic patients, the differences in the survival time related to the level of lesion are by far smaller. Level T1–S5 type A–C lesions survived for a mean of 31.1 years (range 1.1–63.3), whereas all type AIS D patients survived for a mean of 28.9 years (range 13.3–53.1). The Wilcoxon rank sum test was not significant (*p* = 0.4394). The mean life expectancy of ventilator-dependent patients was 12.6 years.

### 3.3. Causes of Death

The following table shows the distribution of causes of death in the study population. Whilst cardiovascular diseases, led by myocardial infarctions and strokes, and pneumonia are leading by far and are responsible for more than 50% of mortalities, it is remarkable that pressure sores are still found in about 10% of cases as a cause of mortality (Table 4).

Table 5 shows the distribution of causes of death depending on the level of lesion and severity of SCI. When investigating the study population, pneumonia was the most frequent cause of death in groups 1 and 2 (50.9% vs. 21.3%), followed by cardiovascular disease (13.2% vs. 19.1%), urosepsis (11.3% vs. 6.4%), suicides (9.4% vs. 10.5%) and malignant tumors (3.8% vs. 21.3%).

Paraplegic patients type T1–S5 AIS A–C died primarily of cardiovascular disease (39.8%), followed by pneumonia (15.3%) and pressure sores (13.3%).

In one of three cases (where the SCI was caused by failed suicide attempts), the cause of death was, again, a new suicide attempt with a fatal end.

Two out of 223 traumatic SCI patients (0.9%) died in a further accident.

Among the 33 tumor patients, 15 suffered from a finally lethal bladder carcinoma (45.5%).

The suicide rate of ventilator-dependent patients was 33.3%, whereas the other groups showed a uniform distribution.

There are two groups in the study population that can be clustered following the insurance status. Those patients that had an accident at their workplace (*n* = 124) and those patients that had their accident during private time (*n* = 99). These types of accidents are remunerated by different insurance systems with differing payment schemes. Patients that sustained an SCI during their occupation were significantly more likely to die of non-SCI related causes (*p* < 0.01, Chi-square test, see Table 6).

The statistically significant connection between the type of insurance and SCI-related causes was identified by a chi-square test (*p* = 0.0344).

## 4. Discussion

The WHO reports a prevalence of 1000/million worldwide [22]. In Germany, the published prevalence of 500/million ranks midfield between Finland (280/million), Norway (365/million) and Canada 1289/million) [1,23,24,25]. International studies of SCI incidence report figures of 13/million (Finland and Germany) and 53/million in Canada [1,23,25]. The most frequent causes of traumatic SCI (TSCI) are road traffic accidents followed by falls and violence. The proportion of traffic-related TSCI varies tremendously in the various regions around the world. This is certainly due to differences in the population density, the volume of traffic and to a regionally very diverse level of road safety [26]. Leisure behavior and sports have an influence on SCI incidence as well. Sports-related SCI varies between 1.7% in Nigeria, 10% in the United States and 14.1% in the Netherlands [8,26]. The data from the present study corresponds to international literature revealing an incidence of sports- and leisure-related causes of TSCI of 1.6%. Patients with SCI, who have endured the vulnerable phase of the first months after discharge to their home environment, can assume a higher average life expectancy [8,27].

The expectation of life for tetraplegic individuals, however, is far lower than for paraplegic patients [8,16,28]. However, one has to consider that a relatively high proportion of suicides has a negative effect on the survival rates of the total population studied. In individual cases, times of survival of more than 40 years can be found. Therefore, the question of whether the reduced lifespan of the relevant individuals is likely to be due to nursing deficits and a restricted participation in normal life is justified. If so, the mentioned causes are avoidable ones.

The cause of death of paraplegic individuals and all AIS D patients becomes more and more similar to those of the average population [8,16]. This correlates well with our finding that, overall, AIS D patients survive longer than those diagnosed with AIS A–C. Ischemic events, malignant tumors and COPD were identified as the most common causes of death of paraplegics in the present study population. Nevertheless, a considerable proportion of these individuals still die as a consequence of the SCI-related problem of pressure sores [15,17]. In the present study, 9.5% of the patients died due to the consequences of this specific medical complication. These SCI-related problems are more likely to occur in patients with complete SCI of the cervical spine and especially in those who are ventilator-dependent, thus explaining the drastically lower life expectancy in those groups than in AIS D patients, who are more likely to live out their life until a regular event like a myocardial infarction due to unrelated factors leads to their death. In patients with SCI of the thoracic spine, differences in life expectancy between AIS D and A–C become insignificant. This leads to the conclusion that SCI-related causes of death are more prevalent in patients with cervical lesions and that our ability to provide sufficient care and prevention for problems occurring with thoracic lesions like urogenital infections is more sophisticated when compared to cervical- and ventilation-associated issues like pneumonia. Patient autonomy must play a major role here, as, for example, even patients with complete SCI of the thoracic spine can usually perform intermittent self-catheterization as opposed to using indwelling catheters, which are generally seen as being associated with more urogenital complications.

Regarding the causes of death in tetraplegic patients, there are still big differences to be observed. The international literature reveals consistently high rates of pulmonary complications as the lethal cause [16,29,30,31,32]. Compared to pressure sores, which can be significantly reduced by measures, pulmonary complications appear comprehensible and partially inevitable, especially in patients with high-level lesions. Accordingly, the present study reports pneumonia as the leading cause of death in tetraplegic patients.

There is evidence in the literature that suicide is often disproportionally the cause of a lethal outcome in tetraplegic individuals [6]. The data of the present study appears to confirm this fact. One has to consider, however, that in a significant number of cases, the original cause of SCI was a suicide attempt in the first place. Analyzing the present study population, it was found that 1/3 of the later suicides had a record of mental illness in their past medical history. This fact evidently influences the rate of suicides. With our results, we cannot differentiate whether the surplus of suicides when compared to a general population is influenced by a higher rate of depression in our population. A further study exclusively discussing the topic of suicidality after SCI has already been initiated.

As we mentioned above, 15 out of 33 tumor patients suffered from a finally lethal bladder carcinoma. Regarding the frequency distribution of tumor diseases in Germany, this is an exceptionally high percentage of patients. In this context, we refer to a new study that proved a statistical correlation between SCI patients and the development of bladder cancer [3,13].

Accidents occurring during work or on the way to the place of employment are separately recorded and documented. This particular documentation is required because the German Statutory Accident Insurance (DGUV) is not only responsible for paying the cost of treatment but may possibly also have to grant a disability pension. We could find a significant difference in the mortality by insurance type, which we cannot currently explain; we have commenced further research to explore if this result occurred due to an internal or external bias or if there are other reasons.

We conclude that patients with SCI of the cervical spine are more likely to die prematurely from SCI-related problems like pressure sores, pneumonia, urogenital infections, suicide and even bladder cancer. The differences with non-SCI patients increase in relation to the level of injury, as the life expectancy of those with cervical injuries was discovered to be lower than in those with thoracic injuries. The same was discovered in relation to the severity of injury. In particular, those patients diagnosed with AIS Type D were discovered to have a higher life expectancy than AIS Type A–C patients with cervical lesions. We therefore deem it necessary to further improve preventative care following rehabilitation, especially in those with severe SCI of the cervical spine.

## Figures and Tables

**Table 1:** AIS-Type (*n* = 223).

AIS-Type	Control Group ± 60 *n*	Cases of Death ± 60 *n*
A	804	159
B	166	10
C	380	40
D	248	14
Sum	1598	223

**Table 2:** Causes of TSCI listed in accordance with the International Classification of External Causes of Injuries (*n* = 223) [19].

Cause of TSCI	*n*	Percentage
Sports and leisure	17	7.3
Assaults	6	2.7
Transport	78	35.0
Fall	118	52.9
Others	4	1.8

**Table 3:** Time of survival in years in accordance with the International Spinal Cord Injury Core Data Set [20,21].

Level of Injury and Severity	Average	Range	*n*
C1–4 AIS A, B or C	17.3	1.1–43.4	53
C5–8 AIS A, B or C	22.3	4.0–51.9	49
T1–S5 AIS A, B or C	31.1	1.1–63.3	98
AIS D at any level	28.9	13.3–53.1	14
Ventilator-dependent	12.6	2.8–34.6	9
Total	25.0	1.1–63.3	223

**Table 4:** Cause of death.

Cause of Death	*n*	Percentage
Cardiovascular diseases	63	28.2
Pneumonia	58	26.0
Pressure sore	22	9.9
Suicide	19	8.5
Other tumor	18	8.1
Bladder cancer	15	6.7
Urosepsis	14	6.3
Other sepsis	6	2.7
Others	8	3.6

**Table 5:** Causes of death according to neurological level and severity of injury (*n* = 223); Abbreviations: Group 1: C1–4 AIS A, B or C; Group 2: C5–8 AIS A, B or C; Group 3: T1–S5 AIS A, B or C; Group 4: AIS D at any level; Group 5: Ventilator-dependent.

Cause of Death	1	2	3	4	5	Total
Pneumonia	50.9%	21.3%	15.3%	14.3%	22.2%	26.0%
Cardiovascular Diseases	13.2%	19.1%	39.8%	57.2%	0.0%	28.2%
Pressure sore	5.7%	12.8%	13.3%	0.0%	0.00%	9.9%
Urosepsis	11.3%	6.4%	5.1%	0.0%	0.0%	6.3%
Other sepsis	0.0%	4.3%	2.0%	0.0%	22.2%	2.7%
Bladder cancer	1.9%	12.8%	7.1%	7.1%	0.0%	6.7%
Other tumor	1.9%	8.5%	10.2%	14.3%	11.1%	8.1%
Suicide	9.4%	10.5%	5.2%	7.1%	33.3%	8.5%
Others	5.7%	4.3%	2.0%	0.0%	11.1%	3.6%
Total	100.0%	100.0%	100.0%	100.0%	100.0%	100.0%

**Table 6:** SCI-related causes of death regarding the type of health insurance.

Causes of Death	Statutory Health Insurance (*n* = 99)	German Statutory Accident Insurance (*n* = 124)
SCI-related	54.5%	40.3%
Non-SCI-related	45.5%	59.7%
Total	100.0%	100.0%

## Data Availability

Data is available from the corresponding author upon reasonable request.
